# A Child With Retroauricular Tenderness: Is It Really Mastoiditis?

**DOI:** 10.7759/cureus.39394

**Published:** 2023-05-23

**Authors:** Alaa Saeed, Rubina Maharjan

**Affiliations:** 1 Pediatrics, Woodhull Medical Center, Brooklyn, USA

**Keywords:** mastoiditis mimicker, acute mastoiditis, case report, retroauricular erythema, acute otitis externa, retroauricular cellulitis

## Abstract

The presence of retroauricular tenderness and erythema has always been closely linked to a diagnosis of acute mastoiditis (AM), a condition that has become less common over the years given the advent of antibiotics and pneumococcal vaccination. However, other etiologies such as retroauricular cellulitis can also present similarly. We present the case of a 10-year-old patient who presented with outward and downward protrusion of the left ear with retroauricular tenderness and erythema and was initially presumed to have a diagnosis of AM and admitted for intravenous antibiotic management. Imaging was negative for any evidence of AM, and in retrospect, the patient was diagnosed with retroauricular cellulitis secondary to acute otitis externa. Being familiar with this differential of retroauricular pain and tenderness can lead to more cost-effective patient care and a different approach with antibiotic management.

## Introduction

Acute mastoiditis (AM) is defined as an inflammation of air cells within the mastoid process. It is often a serious complication of acute otitis media (AOM) in children and most commonly presents with otalgia, fever, and retroauricular swelling and tenderness. The causative organism most commonly implicated is *Streptococcus pneumoniae*. Other less commonly encountered organisms include *Streptococcus pyogenes*, *Haemophilus influenzae*, and *Staphylococcus aureus* [1]. AM has become less common in the United States over the past two decades, despite stricter guidelines for antibiotic treatment of AOM. This can be attributed to widespread childhood vaccination [2]. Another cause of retroauricular tenderness with a different microbial etiology is periauricular cellulitis secondary to acute otitis externa (AOE), which is closely linked to *Pseudomonas aeruginosa*. Although its incidence has not been discussed in detail in the literature, it is an expected and well-known complication of AOE. This difference in microbial etiologies makes the distinction between AM and periauricular cellulitis secondary to AOE important. We report a case of retroauricular tenderness which was initially presumed to be AM but in retrospect was diagnosed as a case of retroauricular cellulitis secondary to AOE.

## Case presentation

A previously healthy 10-year-old male presented to the emergency department (ED) complaining of two days of left ear pain. The pain was severe, felt behind the left ear, throbbing in nature, mildly improving with acetaminophen, and associated with subjective fever. One day following the onset of symptoms, redness and swelling were noted behind the left ear, with visible outward protrusion of the left ear compared to the right ear. At the time, the patient was fully vaccinated apart from the human papillomavirus vaccine and the flu shot for the current season.

**Figure 1:**
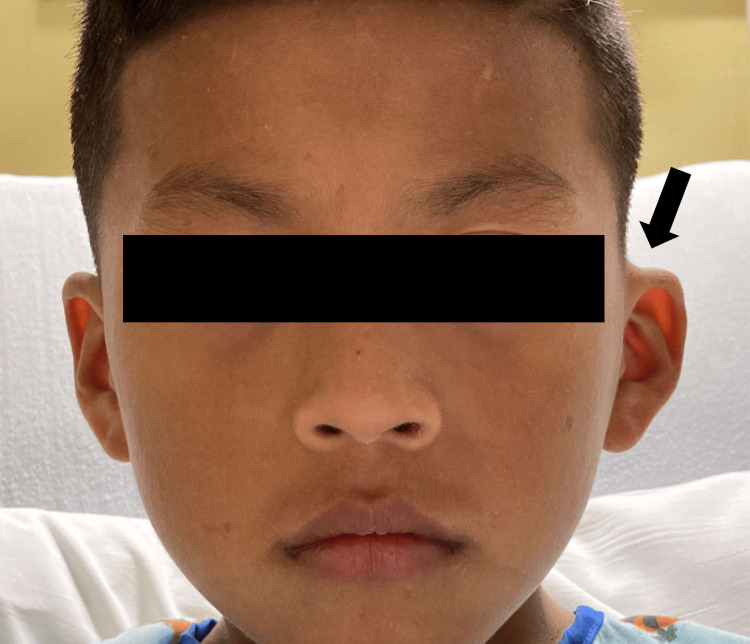
Left ear protrusion.

**Figure 2:**
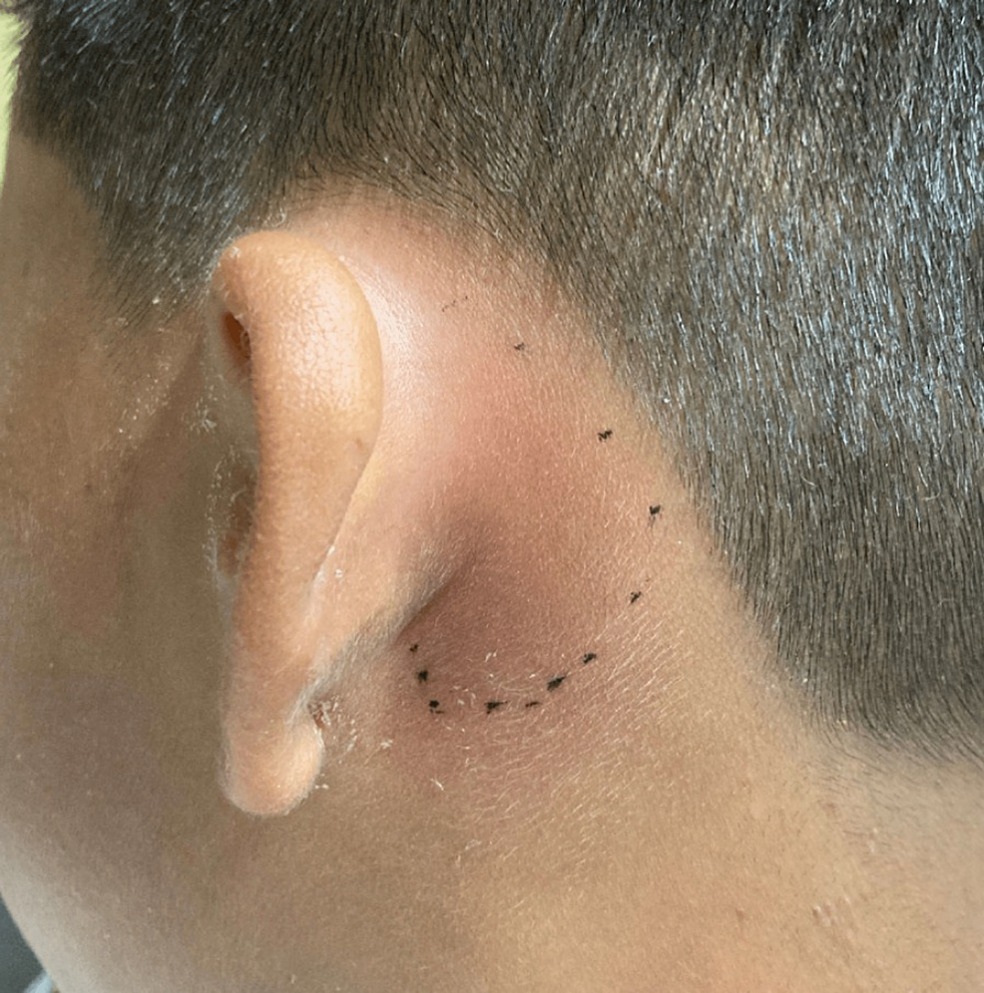
Retroauricular erythema.

In the ED, the patient was afebrile, alert, active, not in acute distress, and with stable vital signs. The left ear was pushed outwards (Figure 1) with retroauricular erythema (Figure 2). On palpation, tragal tenderness in addition to exquisite mastoid tenderness were elicited. The left ear canal was erythematous and occluded with cerumen, limiting the visibility of the tympanic membrane. Examination of the right ear was unremarkable, apart from the impacted cerumen. Initial laboratory workup revealed an elevated C-reactive protein (CRP) (85.52 mg/L) with an unremarkable complete blood count. CT scan of the temporal bone was performed to check for evidence of mastoiditis, and findings were consistent with moderate left supra and retroauricular inflammatory soft tissue swelling without evidence of AM. In addition, associated changes compatible with left chronic otitis media were also reported. Despite the CT scan findings, and due to the lack of pediatric otolaryngology (ENT) services in our setting at that time, it was better to err on the side of caution; thus, it was decided to admit the patient for intravenous (IV) antibiotic management as a case of presumed mastoiditis.

Initially, the patient was started on IV ampicillin/sulbactam, one of the first-line antibiotics for the management of AM. Neomycin-polymyxin-hydrocortisone (Cortisporin) ear drops were also added for suspected co-existing AOE, given the tragal tenderness. Following obtaining a second radiology opinion on the CT scan which confirmed the lack of any features of AM, and due to the radiological evidence of left chronic otitis media, it was decided to change the antibiotic management to IV cefepime to extend coverage to *Pseudomonas aeruginosa*, as well as IV clindamycin to target the causative organisms of cellulitis. Over the course of his hospital stay, the patient remained afebrile, had marked clinical improvement of his symptoms, and had a downtrending CRP. Following five days of IV antibiotics, the patient was discharged on oral levofloxacin, oral clindamycin, and topical Cortisporin drops. The patient was seen by his primary physician four days after discharge and reported no ear pain or fever. There was cerumen impacting the left ear canal, and no retroauricular erythema, swelling, or tenderness. ENT follow-up was not until one month following discharge in a different affiliate facility. At that time, the patient was doing well apart from some left ear discomfort. The impacted cerumen was removed from both ears, revealing normal tympanic membranes bilaterally.

## Discussion

The etiologies causing an acute retroauricular tender swelling in a child are few and tend to include AM as the top differential, followed by cellulitis. Additional etiologies are rarely encountered. Subperiosteal abscess formation in the retroauricular area manifests as a tender fluctuant retroauricular swelling; however, it is usually encountered as a complication of chronic otitis media with cholesteatoma, more often in patients with previous mastoid surgery [3]. Mastoid lymphadenitis can also present with an acute tender swelling, and, very rarely, can even undergo a suppurative process [4].

AM is a condition that has become less common over the years but can present with serious complications if not adequately managed. In the pre-antibiotic era, 20% of cases of AOM were complicated by AM. This percentage decreased with the widespread use of antibiotics and pneumococcal vaccination [1]. The diagnosis of AM is mainly clinical, and the most commonly reported signs include postauricular swelling or fluctuation, erythema, tenderness, and an abnormal tympanic membrane. In addition, the presence of certain risk factors (particularly age <24 months, high CRP, and previous surgical treatment for otitis) may heighten the suspicion of AM [1]. However, to date, there is no consensus regarding specific criteria for diagnosing AM in children [5]. The need for CT scans for uncomplicated cases has been debated in the literature [1], with many centers making it a mandatory part of the initial workup due to the gravity of the complications associated with delay in treatment [6]. On the other hand, some studies recommend limiting CT scans to those with a high risk for complications, the likelihood of an infection with an atypical organism due to previous antibiotic use [6], or recurrent cases. CT findings of mastoiditis usually include opacification of the mastoid cells, loss of sharpness of the mastoid cell walls, haziness of the mastoid outline, and elevation of the periosteum of the mastoid process or posterior cranial fossa [7]. CT scanning is very sensitive but very non-specific, with a study reporting specificity as low as 38% [8], which may lead to overdiagnosis.

AM can be managed pharmacologically or surgically. The mainstay of pharmacological management is antibiotic therapy targeted toward the most commonly implicated organisms, *Streptococcus pneumoniae* and *Streptococcus pyogenes* [6,9]. Given the sensitivity of *Streptococcus pneumoniae* to cephalosporins, an IV third-generation cephalosporin (e.g., ceftriaxone) is the most widely used empiric antibiotic in hospitalized patients with uncomplicated AM following obtaining bacteriologic specimens [1,9,10]. Ampicillin/sulbactam has also been used [7], and clindamycin or metronidazole may be added for additional coverage [9,10]. Antipseudomonal antibiotic coverage is not indicated for uncomplicated AM as it is not a common causative pathogen. Rather, it is an external ear colonizer which can cause misleading false-positive culture results when ear swabs are used as a specimen source. Therefore, the practice of obtaining mastoid cultures or middle ear cultures instead of external ear cultures should be the mainstay of pathogen identification in AM [11].

Combining antibiotic therapy with myringotomy with or without tube placement is important to reduce the rate of complications [10] and decrease the need for mastoidectomy [12]. If no improvement is observed within 24 to 48 hours following the initial treatment, or if any complications are suspected, mastoidectomy should be considered [9]. There is wide variation when it comes to the duration of treatment of AM, and no difference was evident in readmission rates between shorter (<10-14 days) versus longer (>10-14 days) duration of oral antibiotic therapy following discharge [11].

In this era, given how uncommon mastoiditis has become, other differentials of tender postauricular swelling should be considered following careful otoscopic examination. However, literature discussing other etiologies is scant. In a case series, Block demonstrated how periauricular cellulitis secondary to AOE can be a mimicker of AM [13]. Clues include the presence of signs of AOE (severe pain over the tragus and pinna, a swollen ear canal, and canal discharge) with normal plain radiographs and CT scans of the mastoid. Given how AOE is essentially a cellulitis of the ear canal skin and subdermis, its extension beyond the ear canal causing retroauricular cellulitis is not far-fetched. Unlike AM, AOE is most commonly caused by *Pseudomonas aeruginosa* (20%-60% prevalence) and *Staphylococcus aureus* (10%-70% prevalence) [14]. Despite the mainstay of treatment of AOE being topical rather than systemic antibiotics, this is no longer the case when there is an extension of cellulitis beyond the ear canal into the pinna, skin of the neck, or face, as this justifies systemic antibiotics (typically oral and, very rarely, intravenous). Antibiotic coverage should include common AOE pathogens, namely *Pseudomonas aeruginosa* and *Staphylococcus aureus* [14], unlike the case with AM.

In retrospect, despite being overshadowed by the exquisite postauricular tenderness, the presence of hints pointing toward AOE in our patient (tragal tenderness and an erythematous ear canal) along with the absence of evidence of mastoiditis on imaging make the diagnosis of postauricular cellulitis secondary to AOE more likely than the initial diagnosis of presumed AM. This reinforces the importance of a proper ear examination to visualize the tympanic membranes, whether it was a case of AM (for evidence of AOM) or a case of retroauricular cellulitis (for signs of AOE with a normal tympanic membrane). An aural toilet in the ED with the removal of the impacted cerumen (which was likely debris caused by AOE) could have led to visualization of the completely normal tympanic membrane, eliminating the diagnosis of AM. This might have been practically difficult to perform in our setting, given the lack of pediatric ENT services in our center.

## Conclusions

Training pediatric residents on manual removal of impacted cerumen for better visualization of tympanic membranes in patients presenting with suspected AM can aid in clinically ruling out AM, which, in turn, would negate the extra cost and radiation exposure secondary to CT scans, prevent hospitalization, and change the antibiotic management.
